# Impaired placental hemodynamics and function in a non-human primate model of gestational protein restriction

**DOI:** 10.1038/s41598-023-28051-y

**Published:** 2023-01-16

**Authors:** Jamie O. Lo, Matthias C. Schabel, Jessica Gaffney, Katherine S. Lewandowski, Christopher D. Kroenke, Charles T. Roberts, Brian P. Scottoline, Antonio E. Frias, Elinor L. Sullivan, Victoria H. J. Roberts

**Affiliations:** 1 Department of Obstetrics and Gynecology, Oregon Health and Science University, 3181 SW Sam Jackson Park Road, Mail Code L458, Portland, OR 97239 USA; 2 Division of Reproductive and Developmental Sciences, Oregon National Primate Research Center, Oregon Health and Science University, Beaverton, OR USA; 3 Advanced Imaging Research Center, Oregon Health and Science University, Portland, OR USA; 4 Division of Neuroscience, Oregon National Primate Research Center, Oregon Health and Science University, Beaverton, OR USA; 5 Division of Cardiometabolic Health, Oregon National Primate Research Center, Oregon Health and Science University, Beaverton, OR USA; 6 Department of Pediatrics, Oregon Health and Science University, Portland, OR USA; 7 Department of Psychiatry, Oregon Health and Science University, Portland, OR USA

**Keywords:** Experimental models of disease, Outcomes research, Translational research

## Abstract

Maternal malnutrition increases fetal and neonatal morbidity, partly by affecting placental function and morphology, but its impact on placental hemodynamics are unknown. Our objective was to define the impact of maternal malnutrition on placental oxygen reserve and perfusion in vivo in a rhesus macaque model of protein restriction (PR) using advanced imaging. Animals were fed control (CON, 26% protein), 33% PR diet (17% protein), or a 50% PR diet (13% protein, n = 8/group) preconception and throughout pregnancy. Animals underwent Doppler ultrasound and fetal biometry followed by MRI at gestational days 85 (G85) and 135 (G135; term is G168). Pregnancy loss rates were 0/8 in CON, 1/8 in 33% PR, and 3/8 in 50% PR animals. Fetuses of animals fed a 50% PR diet had a smaller abdominal circumference (G135, p < 0.01). On MRI, placental blood flow was decreased at G135 (p < 0.05) and placental oxygen reserve was reduced (G85, p = 0.05; G135, p = 0.01) in animals fed a 50% PR diet vs. CON. These data demonstrate that a 50% PR diet reduces maternal placental perfusion, decreases fetal oxygen availability, and increases fetal mortality. These alterations in placental hemodynamics may partly explain human growth restriction and stillbirth seen with severe PR diets in the developing world.

## Introduction

Maternal malnutrition is a global health epidemic that adversely impacts fetal outcomes and results in long-term health complications in children^1–5^. Both undernutrition and malnutrition have been well known to predispose offspring to diseases later in life, such as hypertension, metabolic syndrome, and obesity^6,7^. In underdeveloped countries, malnutrition is associated with protein-poor diets that lack adequate quantities of essential amino acids, even if adequate calories are consumed. Protein restriction (PR) in mice alters the expression of placental genes encoding regulators of cell growth and metabolism^5^. While few studies have been performed in humans because of the limitations in accurately documenting total protein consumption, it is recognized that low-protein diets during pregnancy will cause infants to be born small for gestational age (SGA)^8,9^. SGA infants are five times more likely to die in the neonatal period and the first years of life^10^. Thus, it is important to identify modifiable risk factors for SGA, such as maternal diet, which can be addressed to help prevent these complications. Existing animal studies^11^ and limited human studies suggest that placental dysfunction is a key contributor to these fetal growth abnormalities, likely because the placenta occupies a central role in facilitating nutrient exchange from mother to fetus.

Our group has previously established a non-human primate model (NHP) of gestational protein restriction^12^ to study the impact of undernutrition, specifically protein deficiency, on placental function and pregnancy outcomes, as this relationship was previously not well understood. The first cohort of pregnant animals in that study demonstrated that a 50% reduction in dietary protein throughout gestation resulted in reduced placental vascular flux rate, detected by contrast-enhanced ultrasound (CE-US), indicative of decreased placental intervillous perfusion, and a 50% rate of pregnancy loss^12^. However, these flux measurements only provide estimates of in-flow velocity to the placental intervillous space, not total blood flow across the placenta, which provides a more comprehensive view of placental vascular function.

Our work with pregnant NHP models enabled the development of MRI-based techniques for comprehensive in vivo placental imaging to characterize maternal blood flow and oxygen exchange between the placenta and the fetal vasculature^13,14^. We have established a modeling framework to quantify the spatial distribution of placental blood flow from individual maternal spiral artery sources. This method was validated in a NHP model that shares developmental ontogeny and placental structure with humans, using dynamic contrast enhanced-MRI (DCE-MRI) with a maternal intravenous injection of a gadolinium-based contrast agent (GBCA) to identify maternal spiral artery sources^13^. Although DCE-MRI is considered the gold standard, and maternal GBCA administration results in minimal fetal exposure in NHP pregnancies^15,16^, uncertainties regarding the safety of these agents for the developing fetus in human patients encouraged us to develop a similar but GBCA-free method for clinical in vivo hemodynamic assessment. Our work has achieved this by extending our MRI approach using a complementary method to characterize placental oxygen perfusion using endogenous *T*_2_* contrast arising from oxy-/deoxyhemoglobin via blood-oxygen level-dependent (BOLD) MRI^14^. This provides a safe alternative to standard MRI contrast agents for future clinical use in human subjects. We have validated our BOLD-MRI technique using DCE-MRI in the NHP model and demonstrated the ability to determine spatial and quantitative characterization of maternal placental perfusion and oxygenation at the level of the spiral arteries^14^.

The NHP model we use has a gestational term and developmental ontogeny similar to humans, including analogous placental structure and function, making this a suitable translational model for human pregnancy studies^17,18^. The benefit of studying NHP subjects is the ability to minimize the various confounders and inter-subject variation found in existing human studies. The present study focused on the effects of maternal malnutrition throughout pregnancy on both placental oxygen reserve and placental perfusion. We hypothesized that gestational protein restriction throughout pregnancy would impair maternal perfusion of the placenta, resulting in decreased placental oxygenation and altered fetal oxygen availability.

## Materials and methods

### Experimental design

Adult rhesus macaques (*Macaca mulatta*) were chronically maintained on either a control chow diet consisting of 26% protein (CON) or a protein-restricted (PR) diet of 17% protein (33% restriction) or 13% protein (50% restriction). All diets were isocaloric and matched for vitamin and micronutrient content, with the protein calorie deficit compensated with additional carbohydrates in the PR diets (TestDiet, St Louis, MO). This study focused on a subset of pregnant rhesus macaques (n = 24) consisting of 8 per group (CON, 33% PR, 50% PR) that underwent advanced imaging studies of the placenta. The generation of pregnancies and details of housing have been previously published^12^. Pregnancies were confirmed by routine first-trimester 2-dimensional ultrasound (GE Voluson 730 Expert, Kretztechnik, Austria) with standard fetal biometry for gestational dating. All animals underwent Doppler-US (D-US) followed by MRI consisting of *T*_2_* and DCE measurements on gestational day 85 (G85) and G135 (term is G168). All protocols were approved by the Institutional Animal Care and Utilization Committee of the Oregon National Primate Research Center, and guidelines for humane animal care were followed.

### Imaging

#### Doppler-US (D-US)

D-US measurements were collected by a sonographer (J.O.L) using image-directed pulsed and color Doppler equipment (GE Voluson 730) with a 5- to 9-MHz sector probe. The lowest high-pass filter level was used (100 Hz), and an angle of 15° or less between the vessel and the Doppler beam was deemed acceptable. Blood flow velocity waveforms were obtained from the proximal portion of the uterine artery (Uta) as previously described^19–21^. Doppler waveform measurements for the umbilical artery were performed and averaged over three cycles using machine-specific software, and the following measurements were obtained: pulsatility index (PI); velocity time integral (VTI); and fetal heart rate (HR)^19–21^. Similarly, UTA was calculated using PI, VTI and maternal HR. The diameter of the Uta was measured using power angiography as previously described^19–21^. The cross-sectional area (CSA) of the vessel was calculated as CSA = π(diameter/2)^2^. Uterine artery volume blood flow (cQ_Uta_) was calculated using the following formula and corrected by maternal weight: cQ_Uta_ = VTI × CSA × HR. For the placental volume blood flow (cQ_UV_), the Doppler waveforms were obtained from the straight portion of the intra-abdominal umbilical vein (UV) as previously described^12,19,22^. The mean velocity (V_mean_) was calculated as 0.5 of the maximum velocity^23^, to estimate laminar flow in a circular vessel. cQ_UV_ was calculated using the formula: V_mean_ × CSA × 60.

#### MRI

Immediately following the ultrasound procedure, MRI studies were performed on an NHP-dedicated 3 T Siemens TIM-Trio scanner (Erlangen, Germany) using a circularly-polarized (CP) transmit, 15-channel receive radiofrequency (RF) “extremity” coil (QED, Cleveland, OH). After localization of the placenta and acquisition of *T*_2_-weighted half-Fourier acquisition single-shot turbo spin-echo (HASTE) anatomic images in the coronal and axial planes, axial 2D multislice spoiled gradient echo (SPGR) images (TR = 418 ms, flip angle = 30°, 256 × 72 matrix, 1.5 mm isotropic spatial resolution) spanning the entire uterus were acquired at six in-phase echo times (TE = 4.92, 9.84, 19.68, 29.52, 36.90, and 44.28 ms) with monopolar readout gradients. Subsequently, 3D SPGR images were acquired in the coronal plane (TR = 9.50 ms, TE = 2.46 ms, 128 × 56 × 44 matrix, 2.5 mm isotropic spatial resolution, flip angles of 3° and 25°), also covering the entire uterus, to allow estimation of *T*_1_ (longitudinal relaxation time) with the variable flip angle (VFA) method^24^.

Immediately after acquisition of VFA data, 150 volumes of 3D SPGR images were acquired for DCE-MRI (TR = 2.00 ms, TE = 0.72 ms, flip angle = 20°, 6/8 partial Fourier encoding in both phase and slab encode directions, elliptical phase undersampling, parallel imaging with GRAPPA (iPAT factor of 2), acquisition time per frame of 3.64 s), with the field of view and resolution matched to the VFA images. Ten baseline images were acquired prior to intravenous injection of a standard dose of 0.1 mmol/kg of gadoteridol contrast reagent (Prohance, Bracco Diagnostics Inc, Princeton, NJ) at a rate of 30 mL/min using a syringe pump (Harvard Apparatus, Holliston, MA). Anatomic and multiecho imaging was performed during expiratory breath holding, achieved by temporarily suspending ventilation, while DCE-MRI data were acquired during ventilated breathing. Physiological monitoring of pulse rate, arterial blood oxygen saturation, and end-tidal CO_2_ partial pressure was performed throughout the imaging study, with no deviations from normal ranges observed in these parameters. Each physiological parameter was recorded at 10-min intervals, and the values reported herein are averages over the final 40 min of the MRI exam, which overlapped the time period in which placental multiecho and DCE-MRI data were collected. BOLD and DCE-MRI analyses were performed as previously described^13,14^.

### Statistical analysis

Data are expressed as mean ± standard deviation (SD). All animals (n = 24) were analyzed, and differences between PR and CON cohorts at G85 and G135 were tested by a one-way ANOVA (GraphPad Prism).

### Conference presentation

Presented orally at the 38th Society of Maternal Fetal Medicine Annual Meetings in Dallas, Texas, January 29–February 3rd, 2018.

## Results

### Gestational protein restriction, pregnancy outcome, and fetal growth parameters

Conception rates were similar amongst the three diet cohorts; all 24 animals became pregnant in each of the CON and PR cohorts. There were no pregnancy losses in the CON cohort, but 1 of 8 pregnancies in the 33% PR group resulted in miscarriage (at gestational day 159), compared with 3 losses in the 50% PR group (at gestational days 104, 146, 153).

Standard fetal biometry measurements were obtained by ultrasound at G85 and G135 as reported in Table 1. Ultrasounds performed at G135 were notable for significantly smaller fetal abdominal circumferences (p < 0.01) in 50% PR animals compared with CON or 33% PR (Table 1). Such differences were not observed earlier at G85. Similarly, maternal weight was significantly lower at G135 in 50% PR animals (p < 0.01). The remainder of the fetal biometry was notable for a slightly reduced biparietal diameter (BPD), head circumference (HC), and femur length (FL) at both gestational ages in 50% PR fetuses (Table 1).

**Table 1:** Fetal biometry and maternal weight.

Parameter	Gestational day 85			Gestational day 135		
	CON (n = 8)	PR 33% (n = 8)	PR 50% (n = 8)	CON (n = 8)	PR 33% (n = 8)	PR 50% (n = 7)
BPD (mm)	28.4 ± 1.5	27.3 ± 1.7	27.5 ± 2.4	44 ± 1.4	44.8 ± 2.5	43 ± 2.9
AC (cm)	8.9 ± 0.2	8.8 ± 0.4	8.3 ± 0.7	13.9 ± 0.7	13.1 ± 1	12.2 ± 1.2**
FL (mm)	18 ± 1.1	15.2 ± 5.8	16.9 ± 1.4	35.8 ± 2.4	35.9 ± 1.4	33.9 ± 4.1
Maternal weight (kg)	7.8 ± 1.4	7.3 ± 0.9	5.9 ± 0.7**	8.1 ± 1.4	6.8 ± 1.6	5.9 ± 0.8**

*BPD* biparietal diameter, *AC* abdominal circumference, *FL* femur length.

One-way ANOVA. Data are means ± SD. **p < 0.01.

### Placental perfusion and oxygenation

By D-US, we demonstrated a significant reduction in cQuv in the 50% PR group compared to controls and 33% PR at both G85 (p = 0.008) and G135 (Table 2, p = 0.0001). There was also a significant difference in cQuta at G135 in the 50% PR animals (p = 0.0001). However, there was no significant difference in the uterine artery and umbilical artery pulsatility indices in any of the treatment groups at both gestational ages (Table 2).

**Table 2:** Doppler ultrasound and dynamic contrast-enhanced MRI measurements of placental function and oxygenation.

Parameter	Gestational day 85			Gestational day 135		
	CON (n = 8)	PR 33% (n = 8)	PR 50% (n = 8)	CON (n = 8)	PR 33% (n = 8)	PR 50% (n = 7)
Uterine artery PI	1.0 ± 0.3	0.7 ± 0.1	0.7 ± 0.2	0.9 ± 0.2	0.8 ± 0.1	0.7 ± 0.1
Umbilical artery PI	2.1 ± 0.4	2.1 ± 0.2	2.0 ± 0.5	1.4 ± 0.3	1.4 ± 0.6	1.2 ± 0.3
cQuta (ml/min)	183.6 ± 70.9	174.1 ± 33.7	133.0 ± 22.9	217.2 ± 59.7	128.0 ± 55.4	87.9 ± 21.2**
cQuta (ml/min/kg)	24.0 ± 9.6	24.1 ± 4.7	22.5 ± 4.7	27 ± 6.0	20 ± 8.9	14.8 ± 2.5**
cQuv	8.3 ± 1.9	7.0 ± 2.9	4.5 ± 0.9**	26.5 ± 5.7	22.0 ± 7.0	14.4 ± 4.5**
Placental volume (ml)^§^	54.7 ± 11.5	72.5 ± 32.1	54.3 ± 19.5	118.6 ± 25.8	131.7 ± 35.3	104.6 ± 21.3
Normalized MRI Placental blood flow (ml/ml/min)^§^	2.1 ± 0.5	1.9 ± 0.5	2.1 ± 0.7	1.2 ± 0.4	1.4 ± 0.5	0.9 ± 0.2*
Median *T*_2_* values	36.1 ± 19.9	53.1 ± 34.3	29.4 ± 6.2*	17.4 ± 4.8	22.9 ± 14.7	13.3 ± 2.1*

^§^Obtained by DCE-MRI.

*PI* pulsatility index, *VT*I velocity time integral.

CSA (cross section of uterine artery) = π(diameter/2)^2^.

Vmean (mean velocity) = 0.5 × maximum umbilical vein velocity.

cQuta (uterine artery blood flow) = VTI × CSA × HR.

cQuta (ml/min/kg) = cQuta adjusted for maternal weight.

cQuv (placental volume blood flow) = Vmean × CSA × 60.

One-way ANOVA. Data are means ± SD. *p < 0.05, **p < 0.01.

**Figure 1:**
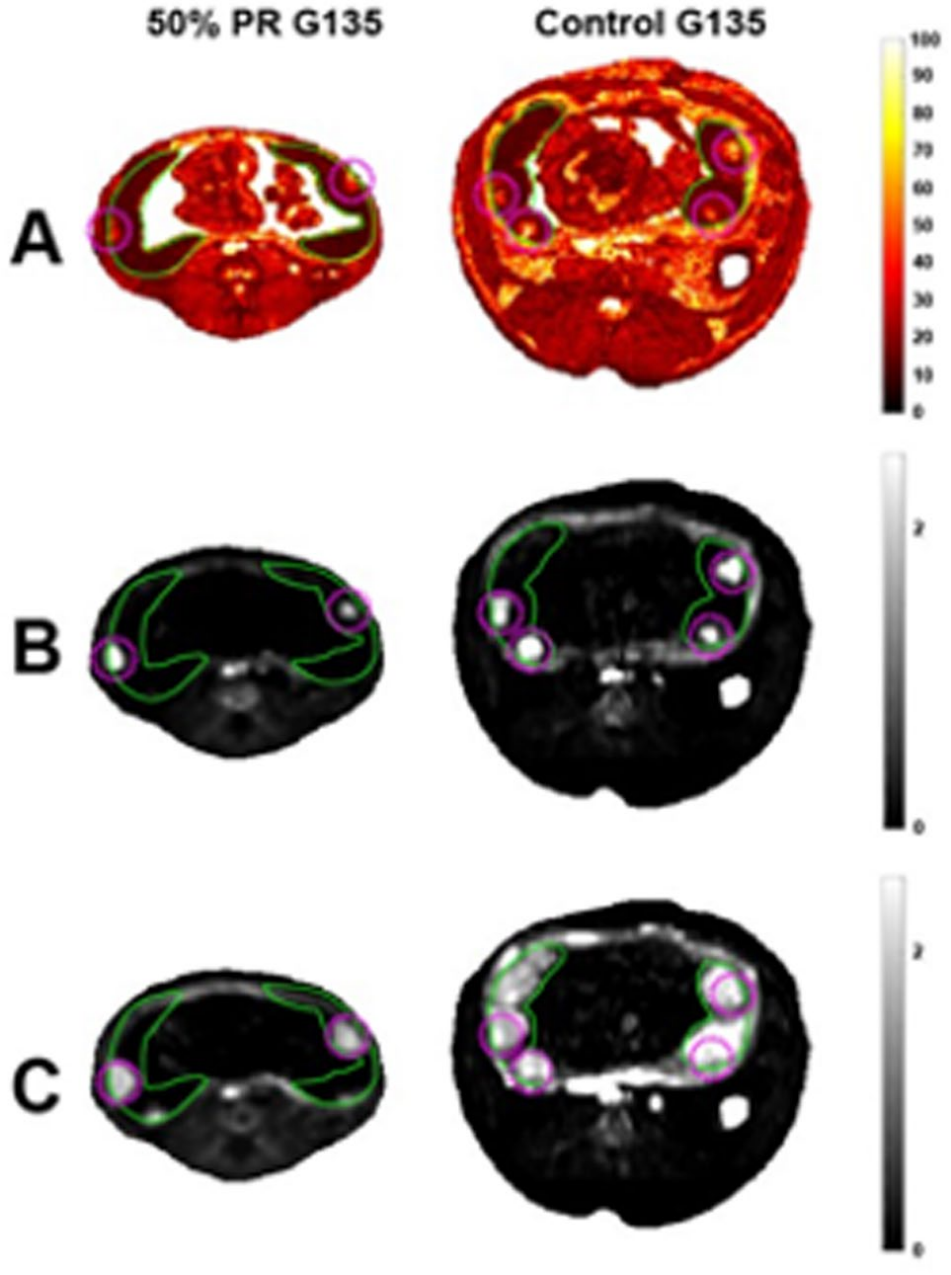
BOLD MRI maps of placental *T*_2_* and corresponding DCE-MRI maps of placental contrast uptake within a single axial slice through the uterus of a representative 50% PR and control animal at G135 with the placenta delineated by the green lines and the spiral artery sources by the magenta regions. (**A**) Maps of *T*_2_* values demonstrate uniformly higher *T*_2_* values in the control placenta relative to those in the 50% PR animal. *T*_2_* values are plotted on a linear color scale from 0 to 100 ms. Placental contrast uptake from dynamic contrast-enhanced magnetic resonance imaging at 30 s (**B**) and at (**C**) 150 s after contrast agent injection. Images display signal change relative to baseline (pre-contrast) signal, and are plotted to emphasize the number and location of spiral artery outlets. In the 50% PR animal, there is avid enhancement near the spiral artery outlets at 30 s, but a substantial portion of the placental tissue is under-perfused, as indicated by the lack of late enhancement 150 s. In contrast, the control placenta enhances more gradually, with multiple sources visible (**B**) and the placenta is nearly completely perfused at the later time point (**C**).

Maternal perfusion of the placental intervillous space was evaluated using DCE-MRI to identify individual spiral arteries and quantify maternal blood flow into the placenta through each of them^14^. This revealed that the total volumetric blood flow to all placental cotyledons, normalized for placental volume, was significantly less in the 50% PR group (p < 0.05) versus CON and 33% PR (Table 2), consistent with the semi-quantitative measures of the cQuta in animals evaluated at G135, but not at G85. Figure 1B,C reflect the substantially under-perfused placenta in a representative 50% PR case compared to a representative control case at two time points following injection of contrast agent.

**Figure 2:**
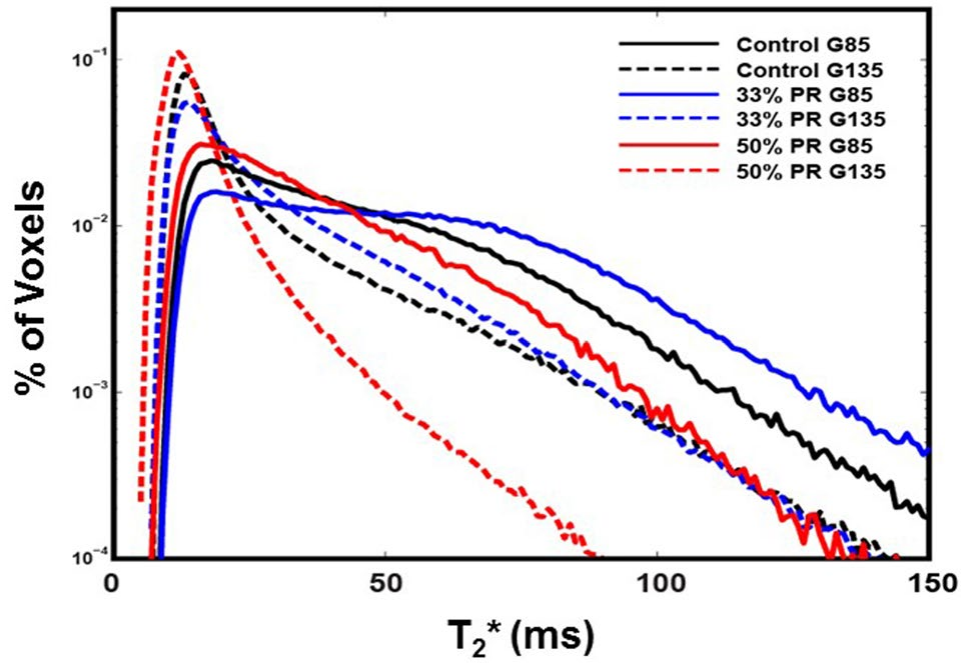
Histograms of placental *T*_2_* for PR vs. control animals at G85 (solid lines) and G135 (dashed lines). 50% PR animals (red) had a significantly smaller fraction of large *T*_2_* values at both G85 and G135 compared to controls (black) and 33% PR (blue), demonstrating decreased fetal oxygen supply in the former group.

Total placental oxygen reserve was determined by analyzing placental *T*_2_* values using BOLD-MRI^14^, which demonstrated significant global reductions in *T*_2_* (blood oxyhemoglobin) in animals fed a 50% PR diet compared with CON and 33% PR at both G85 (p = 0.05) and G135 (p = 0.01) (Fig. 2, Table 2). These findings correlate with decreased placental volume blood flow measured by cQuv at both gestational time points. In placentas of control animals at both G85 and G135, *T*_2_* values were consistent with those observed in control animals in previous reports^14,22^.

The concordance between our DCE-MRI and *T*_2_* measurements is highlighted in Fig. 1. A greater number of spiral arteries are noted on DCE-MRI (Fig. 1B,C) that correlate with observed uniformly longer *T*_2_* values (Fig. 2A) in control animals relative to those in the 50% PR group.

## Discussion

### Principal findings

Using a relevant translational NHP model, our study found that a 50% reduction in dietary protein results in placental insufficiency with decreased maternal placental perfusion, reduced fetal oxygen availability, affected fetal growth, and increased fetal mortality. The finding of diminished maternal placental perfusion is consistent with our reported semi-quantitative findings of reduced placental vascular flux rate by CE-US^12^, and reflects the human condition of maternal malnutrition, in which deficiencies in maternal diet detrimentally impact fetal growth and development^25^. As a functional consequence of the diminished perfusion, fetal oxygen delivery was reduced, as reflected in reduced average placental *T*_2_* in the 50% PR animals compared to controls. However, animals fed 33% less dietary protein were found to have similar placental blood flow and oxygenation as control subjects, suggesting that the placenta has a substantial reserve capacity that can compensate for a certain degree of protein restriction, but not as high as a 50% reduction. Additionally, consistent with our previously reported findings^12^ and other human epidemiological studies^26^, we found a higher incidence of pregnancy loss in the 50% PR cohort.

### Results in the context of what is known

The timing and duration of dietary manipulation is an important consideration for any translational animal model. In our study, protein restriction was initiated prior to conception to reflect a typical human situation in which malnutrition or dietary deficiency is a chronic issue not limited to pregnancy. Prior studies have demonstrated that maternal nutrition prior to conception can largely influence placental and fetal development in the first and second trimester^27^. Previous rodent models studying short-term malnutrition in pregnancy only demonstrated adverse effects of nutrient deficiency on placental growth and fetal development that were dependent on an early versus mid-gestational exposure^28–31^. This is consistent with our observations of significantly decreased maternal placental perfusion and fetal oxygen availability at mid-gestation, G85.

### Clinical implications

As the placenta serves a critical role in maintaining adequate fetal nutrient and oxygen delivery to support normal fetal development^32^, placental dysfunction, as observed in this study, fundamentally impacts fetal growth. We have previously demonstrated the placenta’s ability for compensatory growth and an adaptive increase in the volume of blood flow in response to environmental perturbations in other NHP pregnancy models^22,33^. However, when a critical threshold of its functional reserve is reached, it can result in complications such as fetal growth restriction with increased fetal and neonatal mortality and morbidity^22^. In another sub-study from this NHP model of gestational PR, in which pregnancies were delivered in the mid-third trimester with planned placental tissue studies, we reported evidence of the placenta’s functional reserve capacity^34^. Specifically, placental histological assessment revealed significant accelerated villous maturation, the hallmark characteristic of placental insufficiency, in all 50% PR samples. Additionally, we demonstrated a reduction in placental taurine, an essential amnio acid for fetal development, uptake in an in vitro functional assay, yet minimal impact on fetal body weight in that small sub-cohort of animals^34^. Those data and the findings presented here suggest that the NHP placenta has the ability to compensate for a 33% reduction in maternal protein availability, but that a 50% reduction may exceed the functional reserve and compromises pregnancy outcomes.

### Research implications

The mechanisms underlying the perturbations we observed with significantly decreased placental blood flow and reduced fetal oxygen availability are not well understood. A prior swine study suggested that protein deficiency impairs angiogenesis, resulting in abnormal placental blood flow and amino acid transport^35^. Other low-protein maternal diet sheep and porcine studies reported a decrease of arginine, an essential amino acid for fetal development^36^, in the fetal plasma, allantoic fluid, and placenta^37,38^, as well as impaired placental synthesis of polyamines and nitric oxide, which plays an important role in regulating placental-fetal perfusion and thus, the transfer of nutrients and oxygen from mother to fetus^37–39^. Maternal metabolic adaptations to reduced protein availability are the first consideration in the impact of pregnancy outcomes in any animal model. The ability to grow and develop a placenta with efficient resource allocation will also determine the overall effect on fetal development. Although not yet fully understood, our data suggest that maternal malnutrition from low protein intake significantly diminishes maternal blood flow to the placenta, thereby reducing maternal supply for normal exchange across the placental barrier to match fetal growth demands. This effect may be predicted to compound the effects of reduced maternal substrate, due to PR, available for transfer to the placenta. The reserve capacity of the placenta can buffer some of the potential impacts but is exceeded by severe protein deficiency. We suggest that impaired perfusion and oxygenation underlies placental insufficiency, which impacts fetal growth and, in the most severely affected animals, resulted in stillbirth in our NHP model. This may explain the noted increased risk of human fetal growth restriction and stillbirth with severe gestational protein restricted diets in the developing world.

### Strengths and limitations

This study’s findings highlight the ability of both DCE-MRI and BOLD-MRI as complementary non-invasive imaging techniques to standard D-US of blood flow in major supporting vessels, to comprehensively characterize altered intervillous space perfusion and oxygen exchange with the fetal vasculature in response to an adverse in utero environment. This is an important advantage of the NHP model, in which contrast agents can be used to validate our non-contrast BOLD methodology resulting in MR technology that can be implemented for human use when deemed clinically necessary. Although we were able to detect a significant decrease in cQuta and cQuv by standard D-US that was consistent with our DCE-MRI findings, D-US cannot detect whether protein restriction affected placental oxygen reserve. Indeed, our advanced in vivo MRI method facilitates early identification of pregnancies affected by placental dysfunction, and may have a role in identifying complex pregnancies when delivery timing is unclear by standard clinical evaluation. We have recently demonstrated this potential in a large clinical study of BOLD- MRI across gestation in which we report strong predictive power of this methodology to detect pregnancies at-risk of adverse outcomes^40^.

## Summary

In summary, data from this relevant translational model demonstrated that a 50% gestational PR diet reduces maternal placental perfusion, decreases fetal oxygen availability, and increases fetal mortality. This highlights the importance of preconception and antepartum maternal nutrition, specifically gestational protein intake, on pregnancy and fetal outcomes. Ideally, social policies would be in place to ensure adequate maternal nutrition for women planning to conceive in order to optimize their diet preconception. However, for unplanned pregnancies and those at risk for poor maternal nutrition, our in vivo MRI-based techniques illustrate the need for heightened antenatal surveillance of placental health to facilitate earlier clinical intervention and improve pregnancy outcomes. Our study also further illustrates the translational potential of DCE-MRI and *T*_2_*-based measurements of blood oxygenation, that extends beyond the capabilities of obstetric ultrasound, for detection of pregnancies-at-risk by providing a more detailed evaluation of the in utero environment.

## Data Availability

The datasets used and/or analyzed during the current study are available from the corresponding author on reasonable request.
